# 

**Published:** 1904-05

**Authors:** 


THE AMERICAN

### Journal of Dental Science.

THIRD SERIES.

THE OLDEST DENTAL MAGAZINE IN THE WORLD.

VOL. XXXV. MAY, 1904. NO. 5.

EDITOR:
WM. GIRD BEECROFT, D. D. S., Madison, Wis.
ASSOCIATE EDITORS:
JOHN P. BUCKLEY, Ph. G., D. D. S , Chicago.
ALBERT H. KETCHAM, D. D. S., Denver, Colo.
EMORY A. BRYANT, D. D S., Washington, D. C.
GEO. S MARTIN, D. D. S., L. D. S , Toronto Junction, Ontario.
Publisheu on the 10th day of each month.
Subscription Price, $1.00 per year.
Foreign Countries, $1.50 pe. year.
Address all communications, both business and editorial to the editor.
**Entered as Second Class Matter, Madison, Wis.**

